# Update on occupational allergy, including asthma, to soluble platinum salts

**DOI:** 10.1097/ACI.0000000000000963

**Published:** 2024-02-05

**Authors:** Dick Heederik, Frits van Rooy

**Affiliations:** a Institute for Risk Assessment Sciences, Division of Environmental Allergy; b Arbounie, Expert Center for Chemical Risk Management, Utrecht, The Netherlands

**Keywords:** allergy, asthma, occupational exposure, platinum salts

## Abstract

**Purpose of review:**

This review aims to evaluate recent literature on occupational platinum salt exposure and allergy and asthma in the context of existing evidence.

**Recent findings:**

A major recent development is that large quantitative platinum salt exposure datasets have become available and are finding applications in epidemiological studies. These exposure data are expected to lead to higher quality epidemiological studies focusing on exposure response relations, modifiers of exposure and sensitization risk. The exposure data might also improve medical referral advice as part of medical surveillance studies and contribute to improved evidence on the effectiveness of exposure referral.

**Summary:**

Hopefully, the availability of exposure databases form a stimulus for more exposure response studies and risk assessments leading to science based primary prevention approaches. The availability of more detailed exposure data can guide job transfer decisions in occupational clinical practice.

## INTRODUCTION

The metal manufacturing industry has been associated with a range of pulmonary health risks [1]. Platinum and in particular several platinum salts have been associated with an increased risk of developing allergies including occupational asthma [1,2]. Complex halogenated platinum salts are compounds where the halide is directly coordinated to a central platinum atom [3]. The compounds that contain chlorine, such as potassium tetrachloroplatinate (K_2_PtCL_4_), potassium hexachloroplatinate (K_2_PtCl_6_), ammonium chloroplatinate ((NH_4_)2PtCl_6_) and sodium (hexa)chloroplatinate (Na_2_PtCl_6_) are often referred to as chloroplatinates and are all potent respiratory sensitizers. These salts are water soluble and bioavailable and formed by solubilizing the relatively inert metal form of platinum by strong acids. The bond between the halide group and platinum is weak which facilitates ligand substitution by proteins and subsequent formation of protein antigens [4,5].

Exposure to platinum and its salts occur in the mining and (primary and secondary) refinery industries, and in downstream industries (jewelry, dentistry, pharmaceutical, electrical and chemical industries). In the secondary refining industry, precious metals are reclaimed from scrap material. The most widespread use of platinum at present is use as a catalyst, in particular in the automobile and petrochemical industry. Work processes in catalyst production are highly automated in industrialized countries. Exposure occurs mostly during maintenance and repair.

The first documentation of platinum respiratory disease occurred in 1911 in photographic workers exposed to paper prepared with potassium chloroplatinate [6]. Later, symptoms indicative of asthma, chest tightness, wheezing and shortness of breath, were observed in platinum refinery workers exposed to hexachloroplatinate [7]. It is now well established that platinum workers exposed to the fine dust or mists containing platinum salts frequently develop a syndrome consisting of rhinitis, conjunctivitis and bronchial asthma [2]. Immunological studies in both humans and animals support a type I immunoglobulin E (IgE)-mediated mechanism in patients with hypersensitivity reactions to platinum salts [8]. IgE binds to mast cell and basophils, which release histamine and other active amines after re-exposure. These amines are responsible for development of symptoms which are typical for occupational platinum allergy and asthma. Persistent IgE responses and bronchial hyperresponsiveness may occur even years after removal of exposure.

Exposure to platinum salts occurs in relatively small specialized industries and this explains why platinum salts allergy is not a leading cause of occupational allergy although the risk for exposed workers to develop platinum salt respiratory allergy is relatively high with a cumulative risk higher than 50% over a period within 5 years of exposure [2].

The most recent narrative review can be found in the 5th edition of the book on “Asthma in the workplace” from 2022 [2]. A more exposure oriented peer reviewed overview of the literature published until 2017 on platinum group metal exposure and health effects is also available [9]. Systematic reviews and meta-analyses are lacking. Surveys conducted in the past were relatively small (populations sizes between 10 and approximately 300 subject) with one exception; a study, including 1040 workers [5,7,10–18]. Of the reviewed studies, six were longitudinal studies with a follow-up period between <1 and 30 years. Most studies had a small, often descriptive, exposure assessment component and this limited the possibilities to explore exposure-risk relations. Major conclusions from multiple cross-sectional studies indicate that the prevalence of positive skin prick tests among exposed workers is high, between 14% and 28%. The prevalence seems lower in studies conducted during the last decade of the previous millennium, when exposures were lower than before. Longitudinal studies are indicative of an incidence (in %) or incidence rate (new cases per 100 person years) between 0.5 and 5.

Smoking and atopy are risk modifying factors. There is doubt about the magnitude of the risk modifying effect for instance because selection bias may be present due to preemployment selection for atopy. In addition, not in all studies, exposure and risk modifiers have been mutually adjusted in multiple regression analyses, which may also lead to biased estimates. 

**Box 1:**
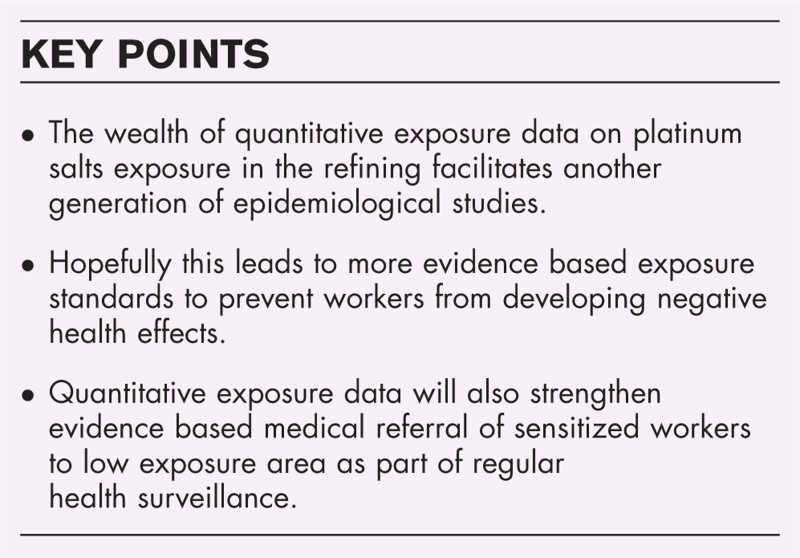
no caption available

## RECENT STUDIES

Smit *et al.*[19^▪▪^] analyzed exposure data involving almost 3000 exposure measurements from five platinum refineries in three different countries (South Africa, UK, USA) over a 17-year period. This is the largest quantitative exposure measurement database available in the platinum refinery industry. Advanced modeling of the data showed that in two of the five participating refineries a statistically significant annual decline in average exposure levels of approximately 10% per year was observed. Such a decreasing exposure trend was absent in the other facilities. Approximately 6% of all 2982 personal exposure measurements taken between 2000 and 2016 exceeded the threshold limit value (TLV) of 2000 ng/m^3^ as established by the American Conference of Governmental Industrial Hygienists in 1963 [20,21]. This TLV was based on limited data, in absence of quantitative exposure–response evidence. Several studies have reported platinum salt sensitization incidence at exposure levels below this TLV [9,13,14]. It has therefore been concluded that “exposure–response relationships observed in workers have not been effectively used for the risk assessment and standard setting of Pt salts” [22^▪▪^]. The Dutch Expert Committee on Occupational Standards (DECOS) recommended a considerably lower health-based occupational exposure limit (OEL) of 5 ng/m^3^, using evidence from the study by Merget *et al.*[14,21]. A total of 63% of all measurements exceeded the latter health-based OEL, indicating that still a high risk for developing platinum salt sensitization exists. These data are available for a large series of job titles and allow detailed exposure response modeling in future studies by combining exposure data with health surveillance information as done earlier [13]. This study also indicated that exposure assessment needs further harmonization [19^▪▪^].

The decrease in chloroplatinate concentrations over the seventeen year period may have gradually led to a lower platinum salt sensitization incidence. Such a conclusion seems warranted analyzing longitudinal studies, conducted over almost a 40-year period [10–14,19^▪▪^].

Part of the data described by Smit *et al.*[19^▪▪^] have been described and used in the context of exposure-sensitization analysis in refinery workers from the five participating refineries. A clear quantitative exposure–response relation was observed for chloroplatinate salts and the incidence of specific sensitization among platinum refinery workers. The exposure–response relation for current exposure is characterized by an initial steep increase in risk starting at low exposure levels in the low ng/m^3^ range, and leveling off at levels of >200 ng/m^3^ for current exposure. An exposure lagging analysis suggested that more recent exposure determines the risk for sensitization more strongly than exposures further back in the past. This study indicates that regular surveillance data, as collected over several years in different platinum industries in different countries, using a common survey protocol, developed and advocated to use as a regular surveillance instruments in the platinum industry can result in highly informative data analyses, in particular when sufficient exposure data are available [3].

This and earlier studies on platinum salt exposure and sensitization focused on the inhalatory route of exposure. However, dermal exposure may also contribute to occupational platinum salt exposure and sensitization. In vitro experiments have shown that Pt salts permeates through intact skin. Permeation through skin of individuals of African origin was significantly higher than through skin of individuals of Caucasian origin [23,24]. Dermal exposure to various halogenated platinum salts causes sensitization in mouse models, but also resulted in lung function changes following respiratory challenge with Pt salts [25]. Interestingly, the skin has a higher permeability for potassium hexachloroplatinate and a higher retention in comparison to potassium tetrachloroplatinate [26^▪▪^], suggesting the risk depends on the specific platinum salt.

Medical surveillance of workers is a well established approach in precious metals refineries and catalyst production plants as a measure to prevent occupational asthma due to platinum salts [3]. The basic concept of surveillance is that workers at risk for developing occupational asthma will be identified by regular medical evaluations involving skin prick testing. It is common practice to transfer sensitized workers from high to low or no platinum salts exposed jobs. Such an approach has been proposed and published by the international platinum industry [3] and is supported by evidence from referral studies [27,28] and a literature review [29]. Limited disease severity at the moment a sensitized worker is identified, and short duration of exposure after sensitization have been established as predictors of a favorable outcome [30–32]. A recent study among 96 German sensitized platinum workers gives insight in the effect of transfer to low or no exposure jobs [33]. Results showed that secondary prevention in subjects with occupational exposure to platinum salts did not prevent workers from developing persistent asthma in the majority of cases although some improvements were observed. It was recommended that removal from exposure should take place immediately after the observation of a positive skin prick test, irrespective of symptoms. Regular monitoring of airborne platinum salt concentrations was not performed by the plants, the included workers originated from.

The European Network for the Phenotyping of Occupational Asthma published a clinical communication on phenotype characteristics of a series of occupational asthma cases caused by platinum salts (*n* = 14) compared to cases caused by other sensitizing agents (*n* = 441). [34^▪^]. Platinum allergy is more often related to IgE-mediated mechanisms

## CONCLUSION

The fact that large exposure dataset have become available opens opportunities to improve the design and analysis of studies focusing on exposure–response relations, the role of host susceptibility factors and modifiers. The exposure data should also be made of use in future transfer studies to improve the quality of referrals and evidence on the effectiveness of an approach that is seen as a key approach to prevention.

## Acknowledgements

*None.*

### Financial support and sponsorship

*None.*

### Conflicts of interest

*Both authors have been involved over the last 5 years in studies supported by the International Platinum Metal Group Association (IPA). The contents of this manuscript, including any opinions or conclusions, are solely those of both authors. The same applies to studies in which the authors were involved and were funded by IPA.*

## REFERENCES AND RECOMMENDED READING

Papers of particular interest, published within the annual period of review, have been highlighted as:

▪ of special interest
▪▪ of outstanding interest
